# Polysaccharide-Based Membrane Biocompatibility Study of *Anacardium occidentale* L. and Polyvinyl Alcohol after Subcutaneous Implant in Rats

**DOI:** 10.3390/ma15041296

**Published:** 2022-02-10

**Authors:** Angelica de Lima das Chagas, Leiny Paula de Oliveira, Mauricio Vicente Cruz, Renato Miranda de Melo, Marina Pacheco Miguel, Katia Flavia Fernandes, Liliana Borges de Menezes

**Affiliations:** 1 Programa de Pós-Graduação em Ciências da Saúde, Faculdade de Medicina, Universidade Federal de Goiás, Goiânia 74605-050, GO, Brazil; angelicalimac@gmail.com; 2 Programa de Pós-Graduação em Ciências Animal, Escola de Veterinária e Zootecnia, Universidade Federal de Goiás, Goiânia 74001-970, GO, Brazil; ly.paula@hotmail.com (L.P.d.O.); marinapacheco@ufg.br (M.P.M.); 3 Departamento de Áreas Acadêmicas II, Instituto Federal de Educação, Ciência e Tecnologia de Goiás, Campus Goiânia, Goiania 74055-120, GO, Brazil; mauricio.cruz@ifg.edu.br; 4 Departamento de Cirurgia, Faculdade de Medicina, Universidade Federal de Goiás, Goiânia 74605-050, GO, Brazil; dr.renatomelo@gmail.com; 5 Laboratório de Química de Polímeros, Instituto de Ciências Biológicas, ICB2, Campus Samambaia, Universidade Federal de Goiás, Goiania 74690-900, GO, Brazil; kfernandes.lqp@gmail.com; 6 Setor de Patologia, Instituto de Patologia Tropical e Saúde Pública, Universidade Federal de Goiás, Rua 235, s/n, Setor Universitário, Goiânia 74605-050, GO, Brazil

**Keywords:** biocompatible, biomaterial, CGP, mouse

## Abstract

Polymeric membranes are a viable and sustainable option for the biotechnology industry from an economic and environmental point of view. In this study, we evaluated tissue response and tolerance to the implantation of a polymeric membrane prepared with cashew gum polysaccharide (CGP) associated with polyvinyl alcohol (PVA). The objective was to characterize the biocompatibility of the CGP/PVA membrane in vivo. Following the evaluation criteria of the ISO 10993-6 standard, we demonstrated that the CGP/PVA membrane showed moderate tissue reaction, with a non-irritating ISO pattern, a thinner fibrous capsule, and a smaller amount of collagen compared to the positive control group. At 30 and 60 days, the membrane presented a similar amount of mast cells to that observed in the negative control group. The data demonstrate that the CGP/PVA membrane presents biocompatibility in accordance with the ISO 10993-6 standard.

## 1. Introduction

Polysaccharides have attracted increasing attention as promising materials for the development of biomedical devices due to their easy availability, non-toxicity, biocompatibility, biodegradability, renewability, and modifiable character [1]. Additionally, the use of natural products for developing new biomaterials offers the advantage of having a wide variety of chemical structures and being environment friendly [2,3].

Cashew gum (CG) is a polysaccharide obtained from the resin of the trunk and branches of *Anacardium occidentale* L., popularly known as cashew, and composed mainly of galactose and arabinose [4,5]. CG is known for its various qualities, such as antimicrobial activity [6,7,8], healing [9], anti-inflammatory [10], immunomodulatory [11], antidiarrheal [12], gastroprotective [13], anti-asthmatic, and anti-diabetic [14], and larvicide-encapsulating agent in the form of nanoparticles [15], and it also presents cytotoxicity in tumor lines [16]. Schirato et al. [9] suggest that cashew gum polysaccharide (CGP) improves the inflammatory stage of the wound healing process.

Despite their biodegradability, the use of natural polymers results in a material with lesser mechanical properties [1,17]. An alternative to solve this problem is the production of a blend, where two or more materials are mixed to form the final product, which may have intermediate properties between the displayed properties of each constituent material. In short, the performance of the blended material may depend on the performance of the constituent materials [17].

Polymer systems obtained by mixing and/or combining different polymers in the form of gels, particles, membranes, and networks have recently drawn the attention of researchers to develop alternative materials with low toxicity associated with high repair efficiency of tissues [1,18,19].

Polyvinyl alcohol (PVA) has been often used for biomaterial production due to its water-soluble, biodegradable, innocuous, noncarcinogenic, and biocompatible character. Moreover, PVA has excellent film-forming and mechanical properties [20,21]. The addition of PVA and CG results in a blend with improved mechanical properties, especially elasticity, and therefore, improves resistance of the final material to handling.

The objective of this study was to produce a membrane with a blend of CGP and PVA and evaluate its biocompatibility in terms of tissue response to the biomaterial in implants in the back of rats. Nowadays, biomaterials are still costly, and their use is limited. Therefore, this study is relevant for the development of low-cost and easy to acquire biomaterials.

## 2. Materials and Methods

### 2.1. Research Facilities

The CGP/PVA membranes used in the study were synthesized at the UFG Polymer Chemistry Laboratory. The animal experimentation procedures were conducted at the Multi-user Center for Animal Production and Experimentation (CMPEA) IPTSP/UFG, and the procedures for making permanent slides and analysis were conducted at the Laboratory of Histotechnics and Innovation LHIn/IPTSP.

### 2.2. CGP/PVA Membrane Production

The extraction of CGP was described by Cruz et al. [20]. The membranes were obtained using the casting technique (solvent evaporation). A 6% (*w*/*v*) aqueous solution of PVA (product number 363138; Sigma Aldrich, São Paulo, SP, Brazil) was prepared by mixing 2% (*w*/*v*) CGP at 60 °C with constant stirring. Subsequently, 5 mL of 0.75 mol/L sodium metaperiodate solution (NaIO_4_) (product number 1853; Dinâmica, São Paulo, SP, Brazil) was added as an oxidizing agent; 10 mL of 1.0 mol/L phosphoric acid solution (H_3_PO_4_) (product number 145; 99% purity, Vetec, São Paulo, SP, Brazil) was added as a catalyst, and 0.5 mg of mannitol (product number 1738; 98% purity, Dinâmica, São Paulo, SP, Brazil) added as a plasticizer. The solution obtained was deposited in acrylic molds at the rate of 1 mL/cm^2^, and solvent evaporation was allowed to take place at 25 °C for 48 h. Dry films were removed from the molds and washed with distilled water until complete removal of residues. The films were dried at 25 °C and stored in hermetic bottles, according to the methodology described by Cruz et al. [21]. Sterilization was performed in a bottle with saline solution in the autoclave.

### 2.3. In Vitro Degradation Test

To evaluate the in vitro degradation behavior of the CGP/PVA membrane, the Hanks balanced salt solution reported by Pourbaix [22] was used. The solution simulates the extracellular environment and has an ionic composition similar to human blood plasma. The solution consists of 8.00 g NaCl, 0.35 g NaHCO_3_, 0.40 g KCl, 0.06 g KH_2_PO_4_, 0.10 g MgCl_2_·6H_2_O, 0.14 g CaCl_2_, 0.06 g Na_2_HPO_4_·2H_2_O, 0.06 g MgSO_4_·7H_2_O, and 1.00 g glucose in 1000 mL distilled H_2_O, pH 7.4. The 9 mm diameter test samples were inoculated in 15 mL of solution at 37 °C ± 0.5 °C and 80 rpm [22,23].

In vitro degradation was monitored through the change in sample weight, before and after immersion, using the digital scale Celtac FA-2104N (Bioprecisa, Sao Paulo, Brazil). Drying was conducted with absorbent paper at room temperature for 60 min. The samples were weighed every five days for 30 days; at day 60, they were kept immersed in the solution throughout the test period. The percentage of weight loss was determined using the following equation:

%W = (W_0_ − W_t_) × 100/W_0_ (1)

where, W_0_ is the initial weight of the dry sample, and W_t_ is the weight of the dry sample at immersion time t. The entire test was performed in triplicate [23].

### 2.4. Fourier Transform Infrared (FTIR) Spectroscopy

FTIR spectra of CGP, PVA, and the CGP/PVA membrane were acquired on a Perkin Elmer FTIR spectrophotometer (Perkin Elmer, Inc., Waltham, MA, USA) using potassium bromide (KBr) discs prepared from powdered samples mixed with dry KBr. Spectra were recorded (16 scans) in the transparent mode from 4000 to 400 cm^−1^ at 4 cm^−1^ resolution.

### 2.5. Ethical Principles and Guideline Requirements

The experimental procedure was approved by the Ethics Committee on the Use of Animals, and the creation of animals and experiments were conducted according to the guidelines of the NIH Guide (National Institutes of Health, Bethesda, Maryland, MD, USA), the care according to the DBCA (Brazilian Guidelines for Care and Use of Animals for Scientific and Didactic Purposes), and the CONCEA Guidelines for Euthanasia Practices. This study was conducted in accordance with the guidelines of the 3Rs program with the aim of reducing the number of animals used during the experiment and minimizing their pain and discomfort (National Center for the Replacement Refinement and Reduction of Animals in Research—NC3Rs). The experimental results were reported according to the ARRIVE (Animal Research: Reporting of In Vivo Experiments) [24] and Planning of Research and guidelines PREPARE (Experimental Procedures on Animals: Recommendations for Excellence) [25].

### 2.6. Experimental Design

Thirty-six male Wistar rats, approximately three-week-old, weighing between 200 g and 250 g, were placed in boxes (two animals per box) with suitable food and water ad libitum. Box changes were conducted every two days using autoclaved wood shavings.

The animals were kept at a temperature of 20–22 °C in a 12 h light-dark cycle. After an adaptation period of 10 days, the animals were randomly distributed into two groups of 18 animals each. Eighteen animals received two treatments on the right and left sides [26]. The SHAM group (control) underwent a false surgical procedure without membrane implantation, and the membrane group (GM) was subjected to a surgical procedure followed by the implantation of a 9 mm diameter CGP/PVA membrane. The other eighteen animals were part of the latex group (GL, positive control) and subjected to a surgical procedure followed by the implantation of a 9 mm diameter natural latex membrane used as a control for promoting an adequately reactive response in the biomaterial compatibility test system [27]. Each group was randomly divided into three subgroups, with six animals each, according to the times of euthanasia and sample collection at 15, 30, and 60 days after implantation [28].

### 2.7. Surgical Procedure and Post-Operative Care

The animals were anesthetized with a mixture of 10% ketamine hydrochloride (90 mg/kg; Ketamine, Agener, São Paulo, SP, Brazil), 2% xylazine hydrochloride (5 mg/kg; Xilazin^®^, Syntec do Brasil, Paraiba, Brazil), and tramadol hydrochloride (1 mg/kg; Tramadol, Teuto, Anápolis, Brazil) intra-peritoneally. Anesthetized animals were placed in a clean box with low light to stimulate sedation. After confirmation of unconsciousness, trichotomy was performed, followed by antisepsis with 2% chlorhexidine (Riohex, Rioquímica, São José do Rio Preto, Brazil) and 70% alcohol. Surgical wound induction was performed with a scalpel (n° 15) from a 1.0 cm linear skin incision in the animal’s dorsal region, then blunt-tip forceps were used for the delicate spreading of the tissues, subcutaneous tissue, and muscular fascia to perform a tunnel on the right and left sides of each animal in the GM/GSHAM groups.

**Figure 1:**
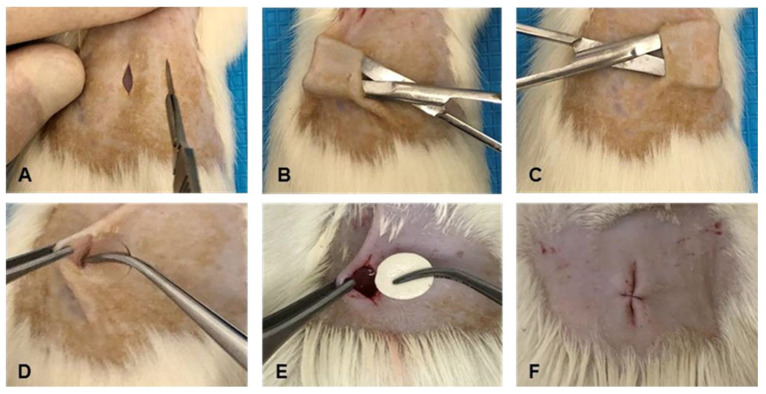
Surgical procedures, Wistar rats. (**A**) Linear 1.0 cm skin incision in the animal’s dorsal region. (**B**,**C**) Divulsion between the subcutaneous tissue and the muscle fascia on the left and right sides. (**D**) CGP/PVA membrane implanted on the left side (GM group). (**E**) Latex membrane implanted on the left (group GL). (**F**) Suture.

The tunnel on the left side received the implant of the CGP/PVA membrane moistened in sterile saline solution (GM group), whereas the tunnel on the right side was not implanted (GSHAM group). In the latex group animals (GL group), a 1.0 cm skin incision was made, and only one tunnel was formed on the left side to accommodate the latex implant. The incision was sutured with 5.0 nylon thread (Ethicon^®^, Johnson and Johnson, San Angelo, TX, USA). The animals were closely monitored until the effects of anesthesia ended. Then, the animals received tramadol hydrochloride (5 mg/kg) diluted in water for three days for analgesia and subcutaneous antibiotic therapy with 10% enrofloxacin (5 mg/kg; Floxiclin, Biofarm LTDA, Jaboticabal, SP, Brazil) for five days [26,29,30]. During the experimental period, the animals were divided into two per cage, separated by an acrylic partition, and examined daily to assess and record any post-operative complications (Figure 1).

At the end of the established treatment period (subgroups of 15, 30, or 60 days), the animals were euthanized in a CO_2_ chamber according to the National Resolution No. 13 of the National Council for the Control of Animal Experiments (CONCEA). After death was confirmed, a necropsy was performed, and samples containing the implant and the adjacent area were collected. The 60-day samples were cleaved, part of the material was used for histological analysis, and another part was used for scanning electron microscopy.

### 2.8. Sample Processing

The samples were subjected to fixation in a 10% buffered paraformaldehyde solution (pH 7.4) for 12 h. After fixation, samples were dehydrated, clarified, and subjected to paraffin embedding. The samples were cut into 4 µm thick sections and stained with hematoxylin and eosin (HE), picrosirius red, or toluidine blue for evaluation under microscopy.

### 2.9. Microscopic Descriptive Analysis

The HE-stained slides were visualized under a conventional optical microscope Leica DM model, coupled with a Leica DFC-280 digital photomicrograph camera (Leica Microssisten, Wetzlar, Germany). A magnification of 40× was used for a more comprehensive view of the area of interest and a 400× magnification for more cell details. For the analysis, five photomicrographs per slide were obtained in non-overlapping fields with 400× magnification. The following aspects were analyzed: amount and type of inflammatory infiltrate at the membrane-tissue interface, neovascularization, presence of giant cells, necrosis, fatty infiltration, granuloma formation, calcification, and degradation pattern of the implanted membrane according to the ISO 10993-6, part 6, Annex E guidelines. Cell counting was performed with ImageJ 1.52 software (National Institutes of Health, Bethesda, MD, USA, Cell Counter plugin).

The thickness of the fibrous capsule was measured using ImageJ software (National Institutes of Health). Five measurements corresponding to approximately the entire implant region were obtained from each slide stained in HE. These measurements were calculated to determine the average thickness of each capsule [31].

For collagen quantification, the samples were stained with picrosirius red and analyzed under a polarized light microscope with a 40× objective. A photomicrograph of five fields per study sample was obtained. Images were captured with a Sony NEX-3 camera (Sony Corporation, Konan, Minato City, Tokyo, Japan) coupled to a Zeiss Axiostar Plus microscope (ZEISS^®^, Burbank, CA, USA) and the collagen quantified as the percentage of the total number of µm using the threshold color tool of the ImageJ^®^ software.

For the quantification of mast cells, samples were stained with toluidine blue. A photomicrograph of five fields was obtained for each sample, and counting was performed with the ImageJ 1.52 software with the Cell Counter plugin.

### 2.10. Evaluation of the Local Biological Effects of the Implantation

#### 2.10.1. Semi-Quantitative Histological Analysis: ISO 10993-6:2016/Part6/Annex E

For each image obtained for HE staining, the histological evaluation was performed based on histopathological criteria of the biological response in each of the six animals investigated in all groups (GM, GL, and SHAM) in the three experimental periods (15, 30, and 60 days). The count of searched elements resulted in a score value that indicated a higher predominance among them. The biological response criteria at the tissue-membrane interface were analyzed and scored as (1) quantity and distribution of inflammatory cells (mononuclear, polymorphonuclear, and multinucleated giant cells), or (2) inflammatory response parameters (neovascularization and degree of fibrosis).

The calculation system followed the guidance of the ISO 10993-6, part 6, annex E. The values of the inflammatory cell infiltrate scores (mononuclear, polymorphonuclear, and multinucleated giant cells) were multiplied by 2 to increase the value in comparison with the parameters of neovascularization and fibrosis. The value was added, and an average for the groups was obtained. The differences between the test groups (GM and GL) and the SHAM group were classified as non-irritant (0.0 to 2.9), mild irritant (3.0 to 8.9), moderate irritant (9.0 to 15.0), and severe irritant (>15).

#### 2.10.2. Scanning Electron Microscopy (SEM)

The structure of the CGP/PVA membrane was analyzed through SEM using a Jeol JSM-6610 microscope (Jeol Ltd., Welwyn Garden City, Hertfordshire, UK) at an accelerating voltage of 5kV. The analyses were performed in the Laboratório Multiusuário de Microscopia de Alta Resolução (LabMic) at the Universidade Federal de Goiás, GO, Brazil.

The analysis of the samples was conducted to characterize the surface morphology under two different conditions: dry and after 60 days of in vivo implantation.

The ultramicrographs from the dry samples were obtained using 500× magnification for surface and 450× transverse. The analysis was performed on the sample fracture surface after maintaining the sample for 5 min at cryogenic temperature, and the surfaces were covered with gold as a conductive material. Selected images were stored.

For observation of the biological samples through SEM, the material (membrane + adjacent tissue) was fixed in 2.5% glutaraldehyde in 0.1 M sodium phosphate buffer, pH 7.2 overnight, and dehydrated in ethanol. The samples were spotted under CO_2_ using the automatic critical point dryer Autosamdri^®^, 815, Series A (Tousimis, Rockville, MD, USA). The analysis was performed on the fracture surface of the sample after maintaining it for 5 min at cryogenic temperature; the surfaces were covered with gold as a conductive material. The photomicrographs obtained from the biological samples were collected at 35×, 300×, and 1000× magnifications. Selected images were stored.

### 2.11. Statistical Analysis

The values obtained from the histological observation of each parameter were arranged in tables using Excel software version 2016 for further statistical analysis, using Prism GraphPad 8.3 software (La Jolla Inc., San Diego, CA, USA). Non-parametric data were calculated using the Kruskal–Wallis test and Dunn’s post-hoc test. A significance level of 5% (*p* < 0.05) was applied to all statistical tests.

## 3. Results

### 3.1. In Vitro Degradation Test

**Figure 2:**
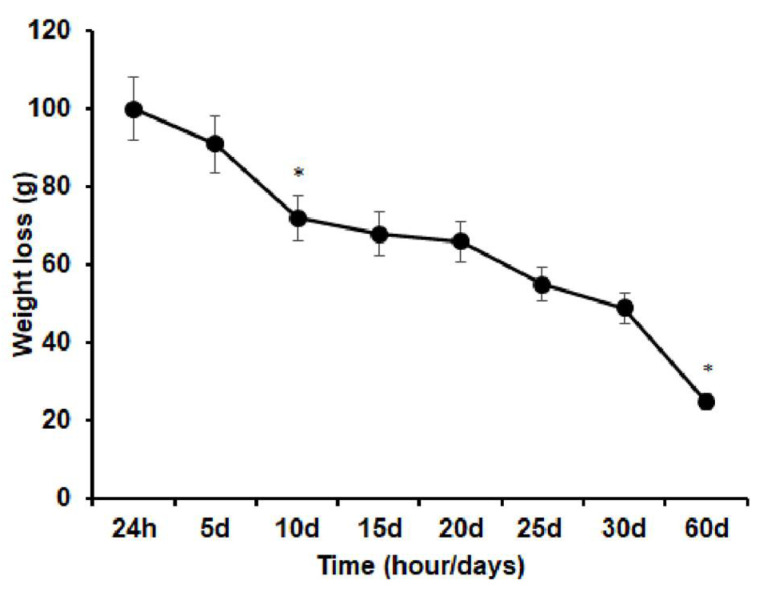
Graphic representation of the means and standard deviations of weight loss of CGP/PVA samples over time after immersion in Hanks’ solution. Non-parametric statistical analysis using the Kruskal–Wallis test and Dunn’s post-hoc test. Significant difference, *p* < 0.05 (*).

To conduct the Hanks solution immersion-induced degradation test, a series of weight change determinations were performed on three CGP/PVA membrane samples. Figure 2 shows the weight reduction within the first 10 days of immersion (*p* = 0.02). Between days 5 and 10 of immersion (*p* = 0.43), a loss of approximately 20% was observed. Membrane weight was stable between days 15 and 20, although with approximately 5% weight loss. At the end of day 60 of immersion, a 45% weight reduction was observed (*p* < 0.01).

### 3.2. Fourier Transform Infrared (FTIR) Spectroscopy

**Figure 3:**
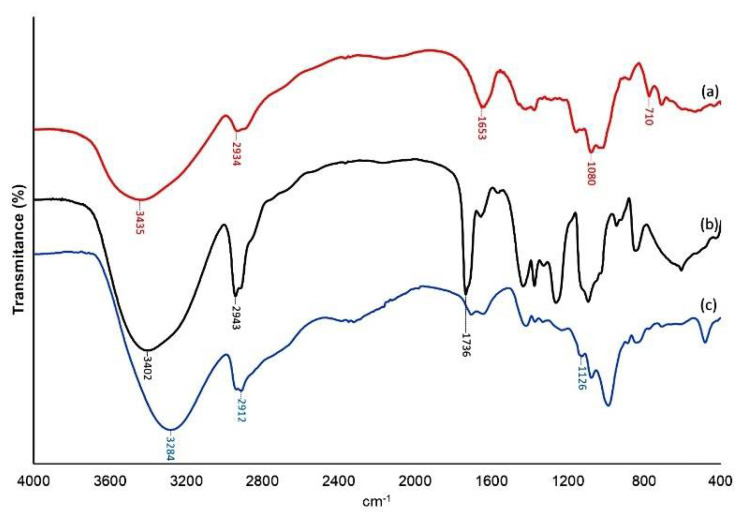
Fourier transform infrared spectra of CGP (**a**), PVA (**b**), and the CGP/PVA (**c**) membrane.

The FTIR spectra of the film components showed the characteristic bands of CGP and PVA. As can be observed in Figure 3a, the CGP spectrum presented a typical band at 1653 cm^−1^, and the PVA spectrum (Figure 3b) showed a typical band at 1736 cm^−1^. After blending CGP and PVA (Figure 3c), the spectrum presented a band 1126 cm^−1^ (Figure 3c).

### 3.3. Scanning Electron Microscopy (SEM)

**Figure 4:**
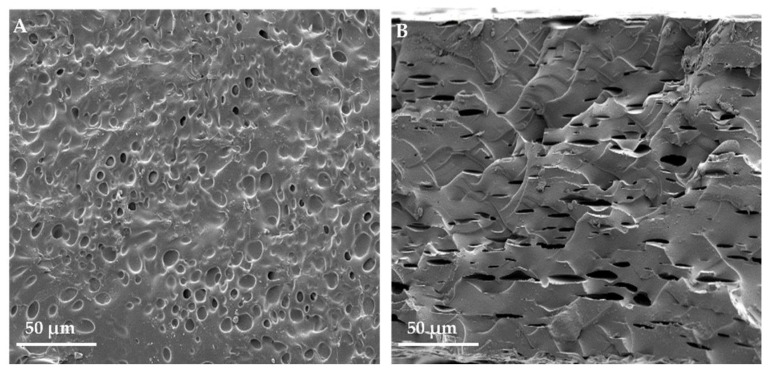
Scanning electron microscopy of CGP/PVA films. (**A**) membrane with a porous surface. (**B**) Cross-section with pores.

Scanning electron microscopy results showed that the dry CGP/PVA membrane had a porous surface (Figure 4).

**Figure 5:**
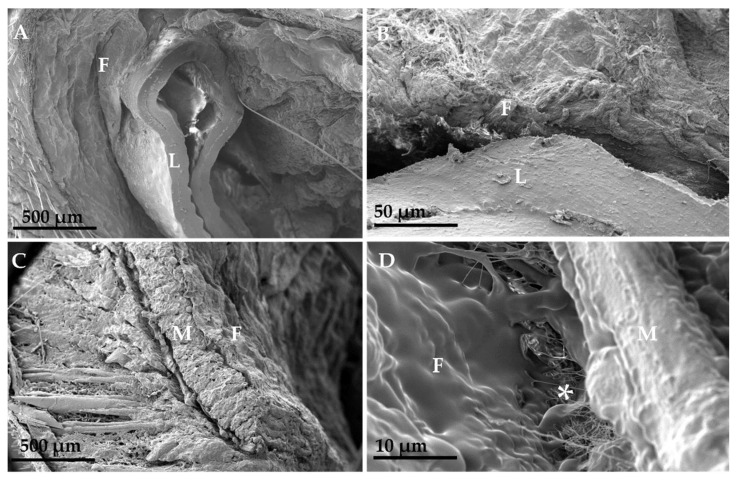
Scanning electron microscopy images showing the membranes (CGP/PVA = M and latex = L) and fibrous capsules (F) that surrounded them at day 60. In (**A**,**B**) (group GL), a dense fibrotic capsule covering the latex membrane, but totally separated from the membrane, was observed. In (**C**,**D**) (GM group), a loosely organized capsule and a clear interaction (*) between the fibrotic capsule and the membrane were observed.

The GL group depicted a dense fibrotic capsule isolating the latex, not showing any point of interaction with the adjacent tissue. The GM group exhibited a uniform layer of connective tissue interaction with the CGP/PVA membrane at the membrane-fibrotic capsule interphase (Figure 5)

### 3.4. Descriptive Analysis of the In Vivo Test

The animals tolerated the surgical procedures well, and the incision healed within seven days with no evidence of infection, ulceration, or tissue discoloration at the implant sites throughout the development of the experiment. During the surgical procedure, some characteristics of the implanted material were identified. As for the handling, the CGP/PVA membrane proved to be a malleable material, resistant, and easy to handle.

At 15, 30, and 60 days, the inflammatory infiltrate was moderate, predominantly mononuclear cells, with the presence of multinucleated giant cells always associated with fragments of membrane degradation, which presented different sizes and shapes. Tissue repair was observed at days 30 and 60 through a reduction in the inflammatory infiltrate, with the presence of neovascularization and connective tissue (Figure 5).

**Figure 6:**
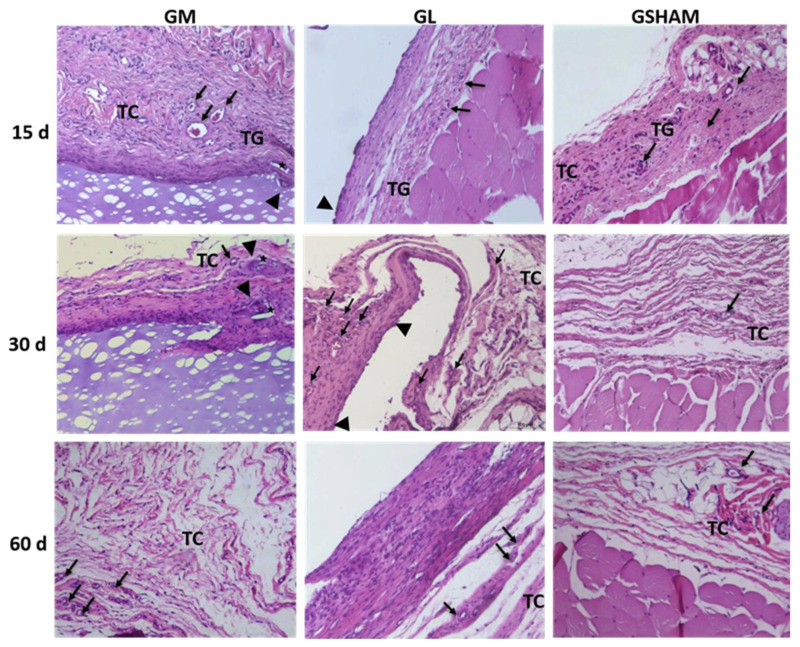
Histopathological analysis of subcutaneous tissue in rats. TG, granulation tissue; TC, connective tissue; (►) giant cell, (**→**) blood vessels, (★) CGP/PVA membrane degradation fragment. Color: Hematoxylin-Eosin. Scale bar: 100 μm.

The GL group showed marked inflammatory infiltrate, predominantly mononuclear cells at all times of the experiment, with a higher amount of inflammatory infiltrate compared to that of the GM and GSHAM groups on the days evaluated and with significant presence of multinucleated giant cells at days 30 and 60 (*p* = 0.0051 and *p* > 0.001), compared to that of the GSHAM group. A gradual increase in the presence of neovascularization was observed, without statistical significance. In the GSHAM group, granulation tissue was observed on day 15, and its replacement by connective tissue, with progressive collagen deposition was observed on days 30 and 60 (Figure 6).

There was no presence of necrosis, fatty infiltration, granuloma formation, or calcification in any of the samples analyzed.

From the semi-quantitative analysis included in the ISO 10993-6 standard, it can be concluded that the GM group (CGP/PVA membrane) presented a biocompatibility pattern classified as non-irritating, with scores remaining below 2.5 during the evaluation period, with a decrease in polymorphonuclear (PMN), mononuclear (MN), and multinucleated giant (MGC) cells over the 60 days of the experiment. The GL group presented a pattern classified as little irritating at all times tested, with values of 5.14, 3.37, and 5.99 at days 15, 30, and 60, respectively (Table 1).

### 3.5. Fibrous Capsule Evaluation

**Figure 7:**
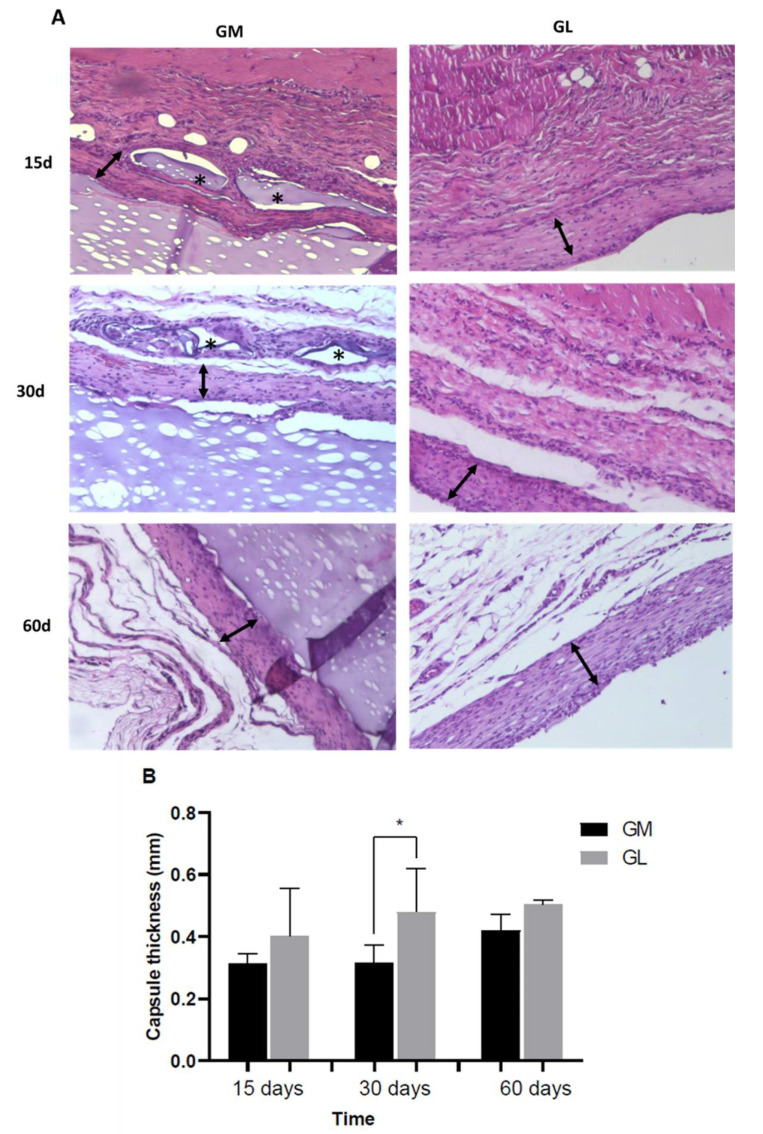
(**A**) Histopathological analysis of sub tissue mouse skin. (↔), capsule thickness; (∗) fragment of degradation. (**B**) Graphic representation of the capsule thickness versus time. Non-parametric statistical analysis using the Kruskal–Wallis test and Dunn’s post-hoc test. Significant difference, *p* < 0.05 (*).

Throughout the period evaluated, the GM group presented a fibrous capsule around the implanted material with interaction in the material–host tissue interface and a gradual increase in thickness in days 30 and 60. No significant differences were observed. The GL group exhibited a fibrous capsule without material–host tissue interaction during the entire period evaluated with a gradual increase in thickness at 30 and 60 days. No statistical differences were observed. On day 30, the capsule in the GL group was thicker (*p* = 0.0333) compared to that in the GM group (Figure 7). In the GSHAM group, no fibrous capsule was observed.

### 3.6. Collagen Assessment

**Figure 8:**
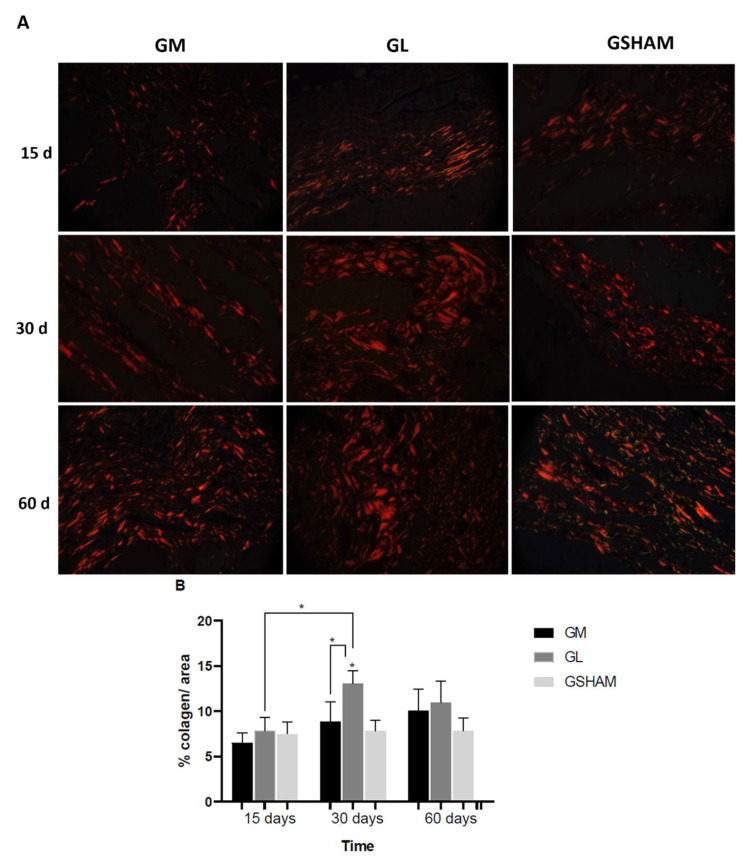
(**A**) Photomicrograph of tissue observed under optical microscopy using polarized light, showing collagen observed in the three experimental groups. Scale bar = 20 µm. (**B**) Quantification of collagen fibers, in area percentage. Non-parametric statistical analysis using the Kruskal–Wallis test and Dunn’s post-hoc test. Significant differences, *p* < 0.05 (*).

In the GM group, the collagen evaluation using picrosirius red staining under polarized light showed red, fine collagen fibers with an increasing decrease in the space between the fibers at the evaluation days (15, 30, and 60 days). In the GL group, collagen showed fine orange fibers, slightly birefringent at day 15, and thick red fibers, with little space between them at days 30 and 60. In the GSHAM group, the collagen followed the same color pattern observed in the GM group: fine red fibers at days 15 and 30 and at day 60, fine reddish and greenish fibers (Figure 8A). The collagen quantitative analysis showed that GM and GSHAM had a lower percentage of collagen when compared to GL; the difference was statistically significant at day 30 after membrane implantation (*p* = 0.0047 and *p* = 0.0224, respectively). The GL group had a higher amount of collagen (*p* = 0.0032) at day 30 compared to days 15 and 60, which was not observed in the other groups (Figure 8B).

### 3.7. Quantification of Mast Cells

**Figure 9:**
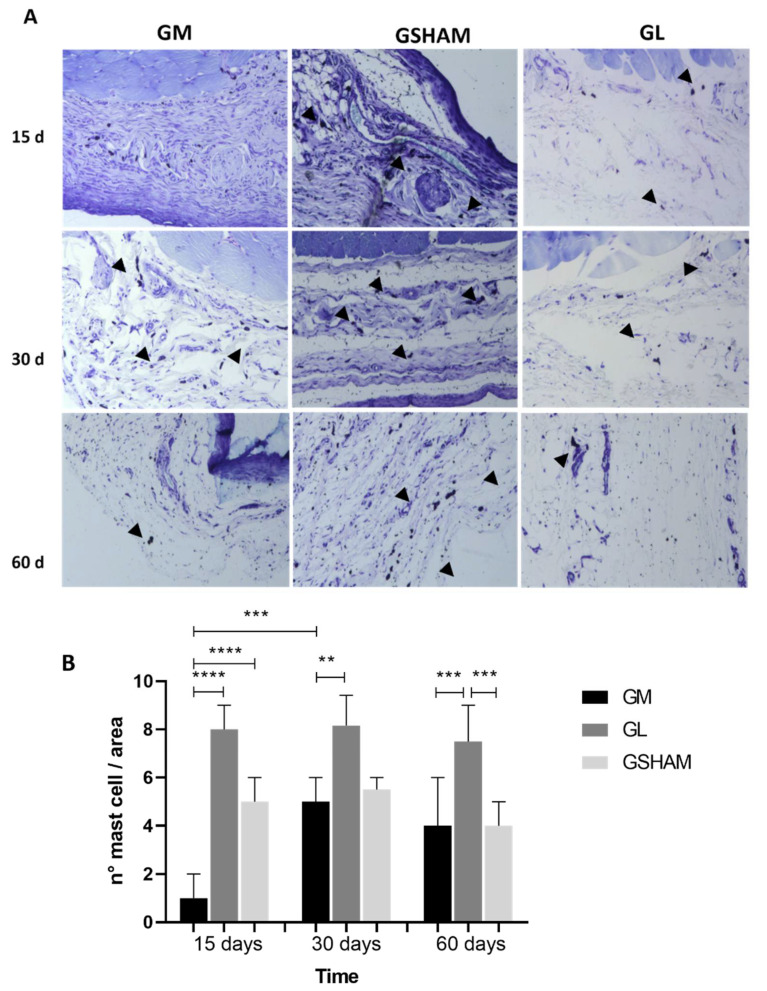
(**A**) Histopathological analysis of rat subcutaneous tissue stained with toluidine blue. Mast cells, (►). Scale bar: 100 μm. (**B**) Graphic representation of mast cell count versus time. Non-parametric statistical analysis using the Kruskal–Wallis test and Dunn’s post-hoc test. Significant differences, *p* < 0.05 (*). (Summary significance <0.0010 **, <0.0005 ***, <0.0001 ****).

On day 15, the GM group had a lower number of mast cells than that in the GSHAM (*p* < 0.0001) and GL (*p* < 0.0001) groups. This observation remained true at days 30 (*p* = 0.0062) and 60 (*p* = 0.0007). On day 60, the GL group still had a higher amount of mast cells when compared to that of the GSHAM group (*p* = 0.0004). The GM group at day 30 showed an increase in mast cells compared to that of the GM group on day 15 (*p* = 0.0007) (Figure 9A,B).

## 4. Discussion

Biocompatible materials are not inert. However, they should not cause an unacceptable physiological response in the host [31,32]. In this study, the subcutaneous implantation method was used to analyze the biocompatibility of the membrane composed of CGP/PVA in rats. This method is considered adequate to assess the response of potential new biomaterials in subcutaneous tissue [33]. Degradation was evaluated in vivo and in vitro; membrane morphology was evaluated through SEM, and biocompatibility assays, cellular reactions, and event chronology were investigated in vivo.

The in vitro degradation test in a solution with physiological characteristics is a method used for evaluating the interaction between the physiological medium and the biomaterial, its stability, and degradation rate. Although it is not possible to completely simulate the chemical characteristics of the living organism, it is an essential process before considering the potential use of a new biomaterial [23]. Although the CGP/PVA film has been studied regarding its composition and properties [20,21], water solubility, and soil burial biodegradability [34], little information on CGP/PVA membrane variations when immersed in simulated body fluid has been reported. Thus, the degradation behavior in Hank’s solution was investigated through the determination of weight loss.

Accelerated degradation is one of the major limitations of polymeric materials [17], as observed by Vicentini [35], who demonstrated losses from 31% to 69% of the initial weight after a week for several polymeric membranes. Silva et al. [34] tested the water solubility and soil degradation of a similar CGP/PVA membrane and observed a solubility of 68% in distilled water at 24 h and a loss of 52% of the initial weight in an open-air burial test at day 90. In the degradation test used in the present study, CGP/PVA membranes degraded considerably slowly in vivo. In the degradation test in vitro, the CGP/PVA membrane showed a 75% loss of the initial weight on day 60, while the mass present in the in vivo test at the end of the 60 days was almost 100%. This result can be explained given the high water solubility of the CGP/PVA membrane [34]. Similarly, Zhang et al. [36] observed a slower in vivo degradation rate of a scaffold prepared with a mixture of natural and synthetic polymers.

There are few reports of polymeric biomaterials that maintain their integrity for 30 days or longer in murine subcutaneous tissue [37,38], although partial or total degradation occurs within 21 days [39,40] or even in 7 days [41]. In this study, the presence of the CGP/PVA membrane was observed within 60 days of implantation. Considering the applicability of the membrane in the medical field, its permanence in a physiological environment for a long time is sufficient to aid in skin repair, which occurs approximately three weeks after the injury [42]. Thus, this characteristic of the material suggests the potential for the biotechnological development of the membrane as a biomaterial with application in wound healing, for controlled release of compounds, for healing, drug delivery, or hormone release.

The FTIR spectra of the film components showed the characteristic CGP, PVA, and CGP/PVA bands. The CGP spectrum revealed a strong, broad band at 3435 cm^−1^ assigned to the stretching vibration of hydroxyl groups of the sugar moieties and a band at 2934 cm^−1^ assigned to the C–H stretching of the alkyl group. It is possible to observe a peak at approximately 1640 cm^−1^ due to O–H scissor vibrations from water molecules bonded to the polysaccharide network. The peaks around 1420 cm^−1^ are related to the symmetric stretching of carboxylic groups (–COO−) from glucuronic acids in the polysaccharide structure. The band interval between 700–1100 cm^−1^, commonly referred to as “fingerprint region”, contains the bands related to the polysaccharide structure. The peaks at ~1080 and 710 cm^−1^ are related to the stretching vibrations of –C–O–C from glycosidic linkages and stretching vibrations of –OH bending from the pyranosidic rings of the sugars present in the CGP structure [43,44,45].

The FTIR spectrum of PVA showed the typical strong hydroxyl bands for free alcohol (nonbonded –OH stretching band at 3600–3650 cm^−1^) and a hydrogen-bonded band (at approximately 3200–3570 cm^−1^) [46,47,48]. A C–H band related to stretching of the C-H alkyl group was observed at 2934 cm^−1^. In addition, the vibrational frequency at 1736 cm^−1^ of pure PVA was assigned to the C–O stretching of the acetate groups in the PVA structure [49]. An important absorption peak was found at a frequency of 1096 cm^−1^. This vibrational band is mostly attributed to the crystallinity of the PVA related to a carboxyl stretching band (C–O, at approximately 1090–1150 cm^−1^) [50,51].

The FTIR spectrum of the CGP/PVA membrane showed the characteristics bands of CGP and PVA (Figure 3c), confirming the formation of the CGP/PVA blend. Two bands were identified as common groups from PVA and CGP: the strong, broad band at 3446 cm^−1^ assignable to the stretching vibrations of the hydroxyl group (from PVA chain and hexoses from CGP) and the band at 2926 cm^−1^ of the CH stretching from the sp3 carbon. The band at 1647 cm^−1^, corresponding to the secondary amine deformations (commonly assigned to primary amino sugar derivatives of the CGP), and the peaks in the region from 1350 to 1400 cm^−1^, corresponding to the folding of CH3 and CH2 groups, were provided by CGP. Strong peaks at 1153, 1081, and 1029 cm^−1^ were related to the stretching vibrations of C-O-C from glucosidic bonds and stretching vibrations of the O-H bending from pyranosidic structures of the sugars or –OH from PVA [43]. Some bands were observed only in the blended membrane, as is the case of the band at 1731 cm^−1^. This band, characteristically assigned to the carbonyl group, allows proposing a reaction route for membrane blended material, involving the hydroxyl group of PVA and carboxyl group from galacturonic acid generated by the oxidative action of sodium metaperiodate. The disappearance of the hydroxyl groups (1600–1650 cm^−1^) and the presence of bands of the acetal ring (C–O–C) and ether (C–O) linkages (1000–1130 cm^−1^) were probably due to the covalent bond formed between oxidized PVA and CGP [43,52].

The homogeneity between polymers and the presence of pores observed through SEM of the membrane are good indicators of the use of the membrane in the preparation of a polymer matrix for tissue remodeling. Surface pores can support cell growth, making the material suitable for use as a tissue framework [1].

The SEM images of the fibrous capsule surface show similar results to those obtained in the histological analysis of the capsule of the different groups, showing a dense and thick mesh in the GL group and a loose and delicate one in the GM group. Some relevant aspects were observed in the SEM of the membrane sections and their fibrous capsules. Unlike the GL group, the GM group showed a capsule in total integration with the membrane through a clear connection layer between them. In the GL group, the material did not show integration with the capsule, and the lack of capsule-membrane integration demonstrated the biological incompatibility of the material.

The surgical implantation of biomaterials triggers a series of reactions in the host. The local reactions involve material-host tissue interaction, inflammatory infiltrate, development of granulation tissue, foreign body reaction, and the formation of a fibrous capsule [31]. The biocompatibility of the implanted material is determined by the intensity of these responses and the ability of tissues to recover after implantation [33,53]. In this study, the GM group showed a milder inflammatory response than that observed for latex (GL group).

The histopathological analysis showed the absence of a non-irritating response of the CGP/PVA membrane according to the ISO standard, which confers biocompatibility to the tested membrane. In vivo studies are considered essential to determine the biocompatibility of any biomaterial. The model proposed by ISO allows the assessment of membrane safety regarding biocompatibility and degradation, assessed by the time the membrane remains in place [54]. Thus, according to the ISO Standard, the present study observed the recruitment of inflammatory cells, the formation of fibrous tissue, and membrane degradation. However, it should be noted that the cell scores of animals with CGP/PVA membranes do not seem to be associated with the physicochemical composition of the membrane, but rather with the natural tissue recovery [30].

Implantable materials are recognized by the body’s defense mechanism, leading to a process known as a foreign body reaction (FBR) with implant encapsulation and the presence of MGC [30,55]. Persistent implants in the tissue stimulate the formation of a fibrous capsule, approximately 50 mm to 200 mm thick, around the implanted material. To isolate the material from the host tissue, its interaction with the adjacent tissue has to be limited, causing the biological response again [56]. In an in vivo experiment with the implantation of several materials, a thicker capsule was observed in the group containing only PVA when compared to groups implanted with other polymeric materials and cotton disks [30]. In this study, the implantation of the CGP/PVA membrane formed a thin fibrous capsule in all the periods analyzed, compared to that in the GL group, diverging from the aforementioned study. In degradable materials, MGCs remain present until the foreign material is fully degraded, while the density of MGCs in non-degradable materials decreases over time [57]. It is important to emphasize that the presence of these cells can occur as an organism attempt to reabsorb the material and does not necessarily imply a lack of biocompatibility.

The size, shape, mechanical properties, type of material, duration, and implantation method determine the type of response in cases of subcutaneous implants, and FBR can be minimized, but not eliminated [55,58]. MGC cells are commonly observed in response to polymeric materials [37] and are present if the material is detected in the subcutaneous tissue [55]. They are commonly considered unwanted since, in the long term, they are the main source of reactive oxygen species, degrading enzymes, and acids that lead to the biodegradation of the implanted material and implant failure [59]. However, this degradation is desired for biodegradable materials [60].

The findings of this study showed the significant presence of MGCs at days 30 and 60 in the GL group. Ibrahim et al. [30] conducted an in vivo experiment implanting several materials, and observed a greater number of MGCs in the group containing only PVA. In this study, the GM group presented giant cells that are related to a significant degradation of the CGP/PVA polymeric membrane at day 60, which may be related to the presence of PVA, as observed in the study by Ibrahim et al. [30]. Thus, the GCP/PVA membrane presents a response similar to those reported in the literature. The presence of MNs and MGCs in GM characterizes a chronic inflammatory response, which may be involved in the biodegradation of the biomaterial, as fragments of membrane degradation were observed.

The use of a special staining and polarized light is more effective in identifying collagen compared to conventional light microscopy [61]. The specificity of the staining is based on the presence of basic amino acids in collagen molecules that react strongly with the acid dye picrosirius red. Such a reaction increases the normal birefringence of collagen, which is composed of aggregated molecules. It is possible to differentiate two types of collagens: type I, strongly refracting with thick reddish fibers, and type III, slightly birefringent and greenish-yellow [62]. In this study, the samples stained with picrosirius red visualized under polarized light showed the predominance of type I collagen fibers. Type I collagen is related to the tensile strength of the newly formed tissue [63]. The type of collagen in GM implants, which has thin red fibers at day 15, may be correlated with a decrease in the space between fibers over time, present in a smaller amount when compared to those of the GL group, and a greater amount when compared to the GSHAM group. The width of the capsule formed in the GM group was less thick compared to that of the GL group, demonstrating a more intense response in GL.

The inflammatory potential was evaluated in terms of the ability of the material to recruit mast cells, which were present in all the samples analyzed. On day 15, GM had a smaller number of mast cells among all tested groups, and a similar amount of mast cells to that observed in the GSHAM group on the other evaluation days. The GL group had a higher number of mast cells throughout the period analyzed. Mast cells participate in the host’s foreign body-type inflammatory response and affect the behavior of fibroblasts and, consequently, the fibrosis process by releasing preformed mediators such as histamine, proteoglycans, proteolytic enzymes, and cytokines [64]. Thevenot et al. [65] observed the absence of fibrous capsules in a subcutaneous implant of biomaterial in rodent knockout for mast cells, concluding that these cells stimulated fibroblast proliferation and subsequent collagen formation, being involved in the regression of the inflammatory reaction and its replacement by fibrous connective tissue. Corroborating the results found in this study, a higher number of mast cells were found in GL, with a thicker fibrous capsule and higher amount of collagen, but without regression of the inflammatory reaction due to its known incompatibility. The GM group had a thinner capsule and a smaller amount of mast cells and collagen, with regression of the inflammatory response over time and was classified as non-irritating according to the ISO standard.

In summary, the results obtained using the ISO irritation pattern, the thickness of the capsule, amount of collagen, and mast cells observed in GM indicate the biocompatibility of the CGP/PVA membrane. The presence of pores, good cell adhesion, biocompatibility, cell-mediated fragmentation and phagocytosis, non-immunogenicity, easy handling, and the slow degradation process observed in GM suggest favorable biological characteristics in the development of membranes for skin wound healing or other biomedical applications that depend on these characteristics.

The biological results of the CGP/PVA membrane tested in this research encourage future studies to produce a membrane for clinical use.

## 5. Conclusions

In this study, the evaluation of the biocompatibility of the CGP/PVA membrane was successfully performed on in vivo implants. This membrane exhibited biocompatibility and low degradability. The presence of a microporous structure in these films allows the material to be explored in several biomedical applications, such as the development of efficient drug delivery systems.

## 6. Patents

Protocol code: BR 10 2020 004173 8.

## Figures and Tables

**Table 1:** Evaluation of implant biocompatibility at days 15, 30, and 60.

Criteria	Scores								
	Day 15			Day 30			Day 60		
	GM	GL	GSHAM	GM	GL	GSHAM	GM	GL	GSHAM
PMN	3.8	4.8	2.2	4.8	4	3.6	2.8	7	1.2
MN	13.2	15.8	4.8	12.6	13.6	9.4	11.8	15.2	6.6
MGC	2	2.8	0.4	1.8	3.4	0.4	1.4	3.8	0
Subtotal (×2)	6.3	7.8	2.46	6.4	7	4.46	5.33	8.66	2.6
Neovascularization	3.6	5	2	4	5.2	2.2	3	4.6	3
Connective tissue	1.6	5.8	10	6	6	4	6	6	8
Subtotal	0.86	1.8	2	1.6	1.86	1.03	1.5	1.76	1.83
Total	7.16	9.6	4.46	8	8.86	5.49	6.8	10.42	4.43
Irritation pattern (test-control) (Teste-control)	2.7	5.14	-	2.5	3.37	-	2.4	5.99	-

Legend: PMN, polymorphonuclear cells; MN, mononuclear cells; MGC, multinucleated giant cell; 0.0 to 2.9, non-irritating; 3.0 to 8.9, slightly irritating; 9.0 to 15.0, moderately irritating; > 15, severely irritating.

## Data Availability

The data presented in this study are available from the corresponding author upon request.
